# Human Umbilical Cord-Derived Mesenchymal Stem Cells Do Not Undergo Malignant Transformation during Long-Term Culturing in Serum-Free Medium

**DOI:** 10.1371/journal.pone.0098565

**Published:** 2014-06-02

**Authors:** Gecai Chen, Aihuan Yue, Zhongbao Ruan, Yigang Yin, RuZhu Wang, Yin Ren, Li Zhu

**Affiliations:** 1 Department of Cardiology, Taizhou People's Hospital, Taizhou, Jiangsu province, China; 2 Jiangsu Beike Bio-Technology Co., Ltd, Taizhou, Jiangsu province, China; French Blood Institute, France

## Abstract

**Background:**

Human umbilical cord-derived mesenchymal stem cells (hUC-MSCs) are in the foreground as a preferable application for treating diseases. However, the safety of hUC-MSCs after long-term culturing *in vitro* in serum-free medium remains unclear.

**Methods:**

hUC-MSCs were separated by adherent tissue culture. hUC-MSCs were cultured in serum-free MesenCult-XF medium and FBS-bases DMEM complete medium. At the 1^st^, 3^rd^, 5^th^, 8^th^, 10^th^, and 15^th^ passage, the differentiation of MSCs into osteogenic, chondrogenic, and adipogenic cells was detected, and MTT, surface antigens were measured. Tumorigenicity was analyzed at the 15^th^ passage. Conventional karyotyping was performed at passage 0, 8, and 15. The telomerase activity of hUC-MSCs at passage 1–15 was analyzed.

**Results:**

Flow cytometry analysis showed that very high expression was detected for CD105, CD73, and CD90 and very low expression for CD45, CD34, CD14, CD79a, and HLA-DR. MSCs could differentiate into osteocytes, chondrocytes, and adipocytes *in vitro*. There was no obvious chromosome elimination, displacement, or chromosomal imbalance as determined from the guidelines of the International System for Human Cytogenetic Nomenclature. Telomerase activity was down-regulated significantly when the culture time was prolonged. Further, no tumors formed in rats injected with hUC-MSCs (P_15_) cultured in serum-free and in serum-containing conditions.

**Conclusion:**

Our data showed that hUC-MSCs met the International Society for Cellular Therapy standards for conditions of long-term *in vitro* culturing at P_15_. Since hUC-MSCs can be safely expanded *in vitro* and are not susceptible to malignant transformation in serum-free medium, these cells are suitable for cell therapy.

## Introduction

The presence of MSCs has been demonstrated in various fetal and adult tissues, including bone marrow, fetal blood and liver, cord blood, amniotic fluid and, in some circumstances, peripheral blood in adults. hUC-MSCs hold great promise as therapeutic agents in regenerative medicine [1],[2]. MSCs from all of the above sources can undergo extensive proliferation *in vitro* and, when cultured under specific conditions, they retain the ability to differentiate into multiple lineages, including bone [3], cartilage, fat, muscle [4]and stromal cells. These cells have attracted considerable interest both because of their value as a model for studying the molecular basis of differentiation and for their therapeutic potential in tissue repair and immune modulation [5].

However, the use of MSCs requires large-scale *in vitro* expansion, which increases the probability of malignant transformation. Some researchers have found that stem cells may naturally undergo malignant transformation during a long culture period[6]. Further, hUC-MSCs may also become carcinogenic after transfection or other modifications. Hence, the safety of these stem cells for clinical applications has begun to be examined more closely. Mouse bone marrow-derived MSCs have been shown to undergo spontaneous transformation after long-term *in vitro* culturing [6],[7]. Thus, for the clinical application of MSCs in different fields of medicine, their biosafety must be carefully investigated through appropriate and sensitive tests. The absence of transformation potential in cultured MSCs must be documented before these cells may be considered for treating patients, particularly immunocompromised subjects, in whom the failure of immune surveillance mechanisms might further favor the development of tumors *in vivo*.

The components of the culture medium are considered to be the main factors affecting the biological characteristics of *in vitro* cultured MSCs. The basic medium composition does not seem significant, and Dulbecco's modified Eagle medium (DMEM) or α-minimum essential medium (α-MEM) can be used for *in vitro* culture of MSCs. However, the two pivotal compounds in the medium that could be responsible for malignant transformation are serum, of either animal or human origin (fetal calf serum (FCS) or human serum or plasma), and growth factors. Classically, the optimal conditions for MSC expansion require FCS-supplemented media, the standard being 10% FCS [8]. The FCS needs to be carefully tested to ensure the best expansion rate. Although FCS may be carefully tested for viruses, the risk of transmission of infectious diseases cannot be excluded. Moreover, in such a culture medium, MSCs retain some FCS proteins in their cytoplasm, which may elicit immunologic responses *in vivo*. Some serum-free media have been developed for the purpose of research, but media suitable for clinical-scale production of MSCs in accordance with good manufacturing practice guidelines have yet to be formulated and characterized. Thus, the aim of the present study was to investigate the potential susceptibility of hUC-MSCs to malignant transformation *in vitro* in serum-free medium and to ascertain whether the biological properties of these cells after expansion remain appropriate for their use in cell therapy.

## Materials and Methods

### Isolation and culture of hUC-MSCs

Ten human umbilical cords were obtained from healthy full-term and naturally delivered newborns after written informed consent was obtained from their mothers and family members. The study protocols were reviewed and approved by the hospital review board and ethics committee of Taizhou People's Hospital. hUC-MSCs were separated using adherent tissue culture. For this, the cord tissue was transferred to a Petri dish containing 20–40 mL sodium chloride using sterile forceps and washed (to remove blood). Wharton's jelly was teased out of the cord and collected in another Petri dish, and the arterial blood vessel, venous blood vessel, and amnion were discarded. The Wharton's jelly was sliced into small fragments around 1 mm in diameter and added to culture flasks. They were covered with glass slides to prevent them from floating. After the hUC-MSCs adhered to the flasks, the medium was changed every 3–4 days. The hUC-MSCs were cultured in serum-free MesenCult-XF medium and and FBS-bases DMEM complete medium (Stemcell, Vancouver, Canada) at 37°C in a humidified atmosphere with 5% CO_2_. At this point, cells were considered to be at stage 0 (P_0_). Once the cells reached 70–80% confluence, they were scraped off using 0.05% trypsin/EDTA and passaged at a density of 1×10^4^ to 0.4×10^6^ cells/cm^2^ [8]. The cells were observed under a microscope and their morphology recorded. The time span from seeding Wharton's jelly to harvesting P_0_ was analyzed in serum-free and in serum-containing conditions.

### Immunophenotyping of hUC-MSCs

hUC-MSCs (1×10^7^cells) were harvested with trypsin and washed twice with PBS. After they were filtered through a 200-mesh screen, the cell concentration was adjusted to 2×10^6^/mL. The surface molecules on the hUC-MSCs were then examined by flow cytometry with the following antibodies: CD34-PE, CD45-FITC, CD73-PE, CD14-FITC, CD79a-APC, HLA-DR-PE, CD90-APC, and CD105-PE.

### Differentiation potential of MSCs

#### Osteogenesis Differentiation

MSCs (6×10^4^ cells/well) were seeded in 24-well plates and cultured at 37°C in a humidified atmosphere with 5% CO_2_ in DMEM/F12 supplemented with a 0.10 volume fraction of fetal bovine serum (FBS), 1.0×10^−8^ mol/L dexamethasone, 2.0×10^−4^ mol/L antiscorbic acid, and 7.0×10^−3^ mol/L β-glycerophosphate. The medium was changed every 3–4 days. After 21 days of culture, the cells were processed for Alizarin Red S staining, to detect osteogenesis. The cells were fixed in 70% ethanol for 1 h at room temperature, washed with PBS, stained with 40 mM Alizarin Red S (pH 4.2) for 10 min at room temperature, washed five times with deionized water, and incubated in PBS for 15 min to eliminate non-specific staining. The stained matrix was then observed at different magnifications under a microscope [9].

#### Adipogenesis Differentiation

MSCs (6×10^4^ cells/well) were seeded in 24-well plates and cultured at 37°C in a humidified atmosphere with 5% CO_2_ in DMEM/F12 supplemented with a 0.10 volume fraction of FBS, 1.0×10^−6^ mol/L dexamethasone, 10 mg/L insulin, 100 mg/L 1-methyl-3-isobutyl xanthine, 100 mg/L indomethacin, 100 U/mL penicillin, and100 mg/L streptomycin_._ This adipogenesis differentiation medium was replaced every 3–4 days. After 14 days of culture, the cells can be processed for Oil Red O staining, to detect adipogenesis. The staining process was similar to that used for detection of osteogenesis.

#### Chondrogenic Differentiation

For this test, 7.5×10^5^ MSCs were seeded in 6-well plates and cultured at 37°C in a humidified atmosphere with 5% CO_2_ in DMEM/F12 supplemented with 0.10 volume fraction of FBS, 1.0×10^−7^ mol/L dexamethasone, 6.25 mg/L insulin, 50 µg/L vitamin C, 6.25 mg/L transferrin, and 10 µg/L transforming growth factor β. This chondrogenic differentiation medium was changed every 3–4 days. Chondrogenic pellets were harvested after 28 days of culture. The pellets were formalin-fixed and paraffin-embedded for Alcian blue staining.

### Growth curve analysis using the 3-(4,5-dimethylthiazol-2-yl)-2,5-diphenyl tetrazolium bromide (MTT) assay

Proliferation of hUC-MSCs was assessed on days 1, 2, 3, 4, 5, 7, and 8 after initiation of growth, by mitochondria-dependent reduction of MTT (Beyotime, Shanghai, China). Cell monolayers were washed twice with two kinds of medium. MTT (100 µL of 5 mg/mL MTT in PBS; final concentration, 0.8 mg/mL) was then added to each well and the cells incubated at 37°C for 6 hours before overnight solubilization in 500 µL sodium dodecyl sulfate (10% wt/vol in 0.01 M HCl). A sample (150 µL) from each duplicate well was then transferred to a 96-well microplate and the optical density was determined using an automated dual wavelength microplate reader against a reagent blank, sample containing all components except for the cells, at a test wavelength of 492 nm and a reference wavelength of 630 nm. Growth curve analysis by MTT assay at P_8_ of MSCs from the ten donors in two kinds of conditions. Data shown are the mean ± SEM of values for the ten donors.

Population doubling time(PDT) was calculated by the following formula:

PDT =  (CT x ln2)/ln (Nf/Ni), where CT is cell culture time, Ni is the initial number of cells and Nf is the final number of cells.

### Telomerase activity assay

Using a telomerase detection kit (TRAPEZE Telomerase Detection Kit, Chemicon, S7700), the telomerase activity of cultured MSCs cells was detected and quantified using a software program [10],[11].

### Karyotype analysis

For this analysis, 2×10^6^ cells were harvested and 0.1–0.4 µg/mL colchicine was added to the culture medium. The cells were collected after 12 h, and 0.075 M KCl was added to them, after which they were placed in a 37°C water-bath. Next, 1 mL fixative (methanol/acetic acid mixture at 1∶3) was added to the cells, and they were incubated for 30 min at 37°C. The cells were centrifuged, and collected. Then, 8 mL fixative was added, and the cells were again placed in the 37°C water-bath for fixing; subsequently, they were dyed for 10 min with 10% Giemsa stain, washed with distilled water, and observed under an electron microscope after drying at room temperature. Chromosome analysis was carried out by using G-bands following the guidelines of the International System for Chromosome Nomenclature 2009 (ISCN 2009). On average, 20 metaphases were evaluated, and cells from passage 1 and 15 were tested. If the karyotype of passage 0 cells was abnormal, the sample was discarded.

### Tumorigenicity test

Healthy, 4-week-old male nude mice were provided by the Animal Center of Second Military Medical University. The procedure for the animal experiment was approved by the Institutional Animal Care and Use Committee at the Animal Center of the Second Military Medical University. *In vitro* tumorigenicity studies were carried out with the blank controls, hUC-MSCs in two culture medium (P_15_ from ten donors were pooled), and SGC7901 human gastric cancer cells. For this, 40 nude mice were randomly divided into five different groups. Next, 1×10^8^ cells were suspended in 2 mL physiological saline, and 0.2 mL of this cell suspension was injected subcutaneously into the right forelimb of each nude mouse. The control group was administered 0.2 mL saline. Subcutaneous tissue from the armpit of the mice were stained with hematoxylin-eosin.

### Statistical analysis

Data were expressed as mean +/− SEM. Comparisons of mean values among the passages were analyzed using a Tukey's multiple comparison test. “Serum-free” vs “serum-containing” were compared using Student's t test. A five percent probability (*P<0.05*) was used as the level of significance. Differences were considered as statistically significant with *P<0.01*.

## Results

### Morphologic observation

**Figure 1:**
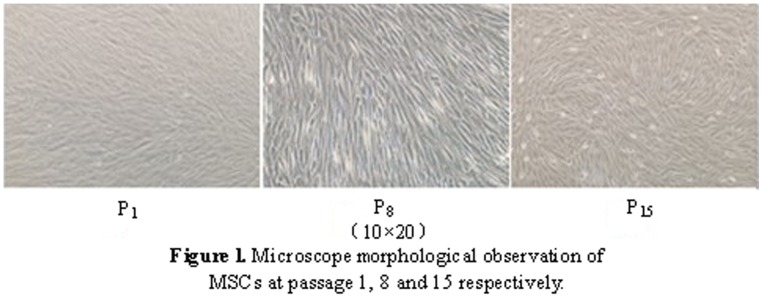
Microscope morphological observation of MSCs at passage 1, 8, and 15 from in serum-free condition.

Morphologic observation of MSCs is the most intuitive method for clearly differentiating cell types. We examined every cell passage of cells from the 10 donors and found no typical morphological changes. Figure 1 shows the morphological results of MSCs derived from donor 2. There was no visible difference in morphology of MSCs between donor 2 and other donors. But the time span from seeding Wharton's jelly to harvesting P0 was different between different donors in the same culture medium. Donor 2 displayed longer time span, compared to one from donor 5 (*P<0.05*).The time span of serum-containing conditions was shorter than serum-free cultured ones (*P<0.05*).

### Immunophenotype analysis by flow cytometry

**Figure 2:**
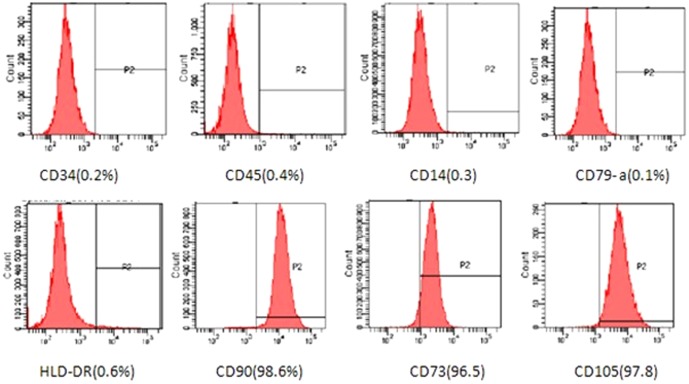
Analysis of MSCs for the expression of surface markers by flow cytometric analysis. The immunofluorescence analysis of MSCs was conducted at the 15^th^ passage of cells obtained from donor 3.

We investigated the immunophenotypes of the MSCs using immunofluorescence flow cytometry, since this is a very important biochemical method used to understand cell types. We tested passage 1, 3, 8, 10 and 15 cells. As shown in Figure 2, very high expression was detected for CD73, CD90, and CD105, and very low expression was detected for CD14, CD45, CD34, CD79a, and HLA-DR. There was no visible difference in immunophenotype of MSCs between 10 donors in two conditions. These results meet the International Society for Cellular Therapy (ISCT) criteria for MSC definition (Figure 2).

### Multilineage potential

**Figure 3:**
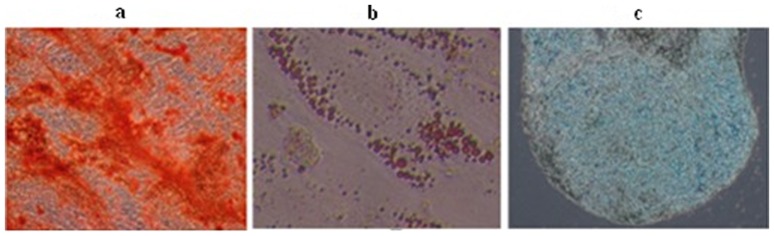
Differentiation of hUC-MSCs from donor 3 was showed at the 15^th^ passage. (a) Alizarin Red S staining to detect osteogenesis differentiation. (b) Oil Red O staining to detect adipogenesis differentiation. (c) Alcian Blue staining to detect chondrogenic differentiation.

An important characteristic of MSCs is their differentiation into osteoblasts, adipocytes, and chondroblasts. To investigate the feasibility of inducing the differentiation of hUC-MSCs into osteoblasts, adipocytes, and chondroblasts *in vitro*, we induced cells from passage 1, 3, 8, 10, and 15 under different culture conditions. We found that all the samples kept multipotent differentiation potential according to ISCT standards (Figure 3). In serum-free and in serum-containing conditions hUC-MSC have comparable differentiation potential from different donors.

### Growth curve analysis by MTT assay

**Figure 4:**
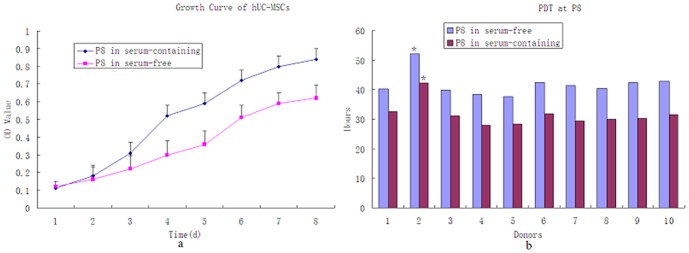
The proliferation capacity of hUC-MSCs : (a).Growth curve analysis by MTT assay at P_8_ of MSCs from the ten donors. Data shown are the mean ± SEM of values form ten donors. (b). The PDT of hUC-MSCs at P_8_ from donor 2 was different from the one from donors 5(*P<0.05*) in the same culture medium. The PDT of serum-containing conditions was shorter than serum-free cultured ones (P<0.05)

hUC-MSCs exhibit robust proliferation properties *in vitro*. The cells were in the latent period during the first couple of days after incubation and proliferation was not evident. Three to seven days into the logarithmic phase, cell proliferation was accelerated, and after 7–8 days the growth plateaued. There were two cell multiplication cycles, one was between the days 3–4 and the other was between days 4–7. The PDT of hUC-MSCs at P_8_ from donor 2 was different from the one from donors 5(*P<0.05*) in the same culture medium. The PDT of serum-containing conditions was shorter than serum-free cultured ones (*P<0.05*). (Fig. 4)

### Quantitative assay for telomerase activity

**Figure 5:**
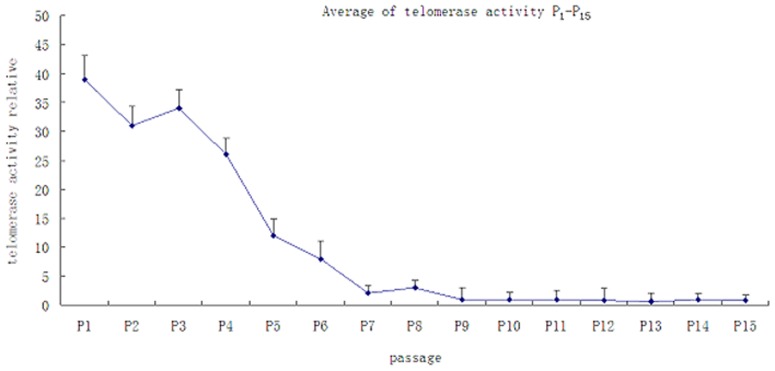
Quantitative assay for telomerase activity of hUC-MSCs at passage 1–15.

To investigate the effects of long-term *in vitro* culture on the genotype of MSCs, we examined telomerase activity. We found that telomerase activity was significantly down-regulated as the culture time increased. The telomerase activity in the MSCs gradually decreased after numerous passages were cultured *in vitro*. The calculations are based on the average values of the 10 donors for each passage (Fig. 5).

### Karyotype analysis

**Figure 6:**
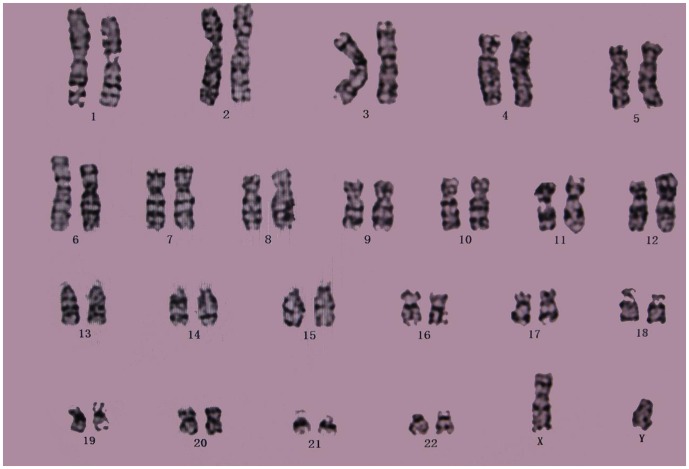
A conventional karyotype analysis performed at the 15^th^ passage (46,XY). The MSCs expanded *in vitro* did not show chromosome elimination, displacement, or imbalances.

Cytogenetic karyotype analysis was performed on cells at passage 0, 8, and 15. As shown in Figure 6, no abnormal chromosome phenomenon was found. Further, there was no chromosome elimination, or displacement or chromosomal imbalances, as determined according to the International System for Human Cytogenetic Nomenclature (Fig. 6).

### Tumorigenicity test

**Figure 7:**
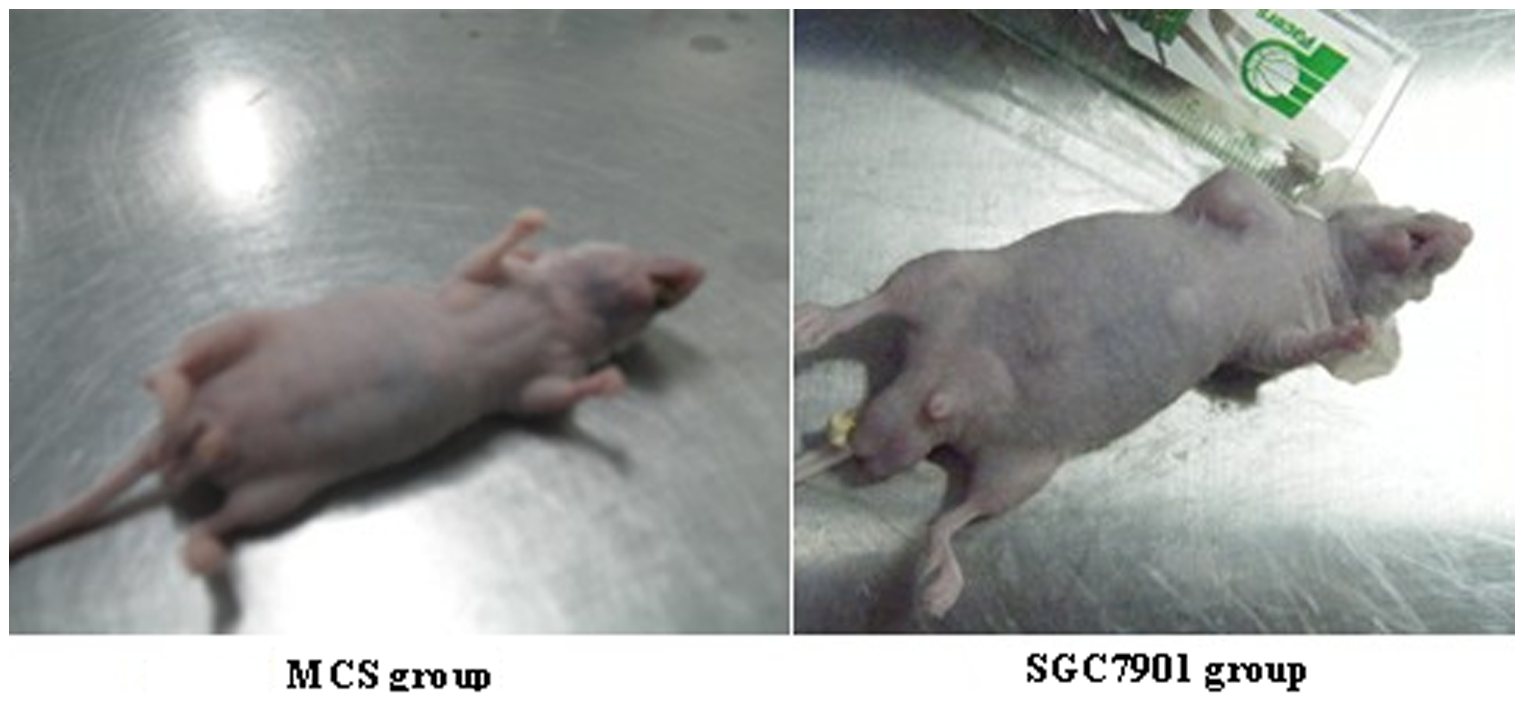
The hUC-MSC group (passage 15) of nude mice showed no tumor formation 4 weeks after the cells were implanted. However, the SGC7901 group developed tumors after 4 weeks.

Finally, we carried out a tumorigenicity assay. We compared tumor formation in mice injected with cells (P_15_) of two culture conditions. After three months of normal feeding, no solid tumor formation was noted in the hUC-MSC (P_15_ from both conditions) or control group. The mice remained in the healthy survival mode and were followed up for 6 months. Hematoxylin-eosin staining showed subcutaneous tissue from the hUC-MSCs group were normal. However, 8 of the 10 mice in the SGC7901 group developed tumors after 3 weeks, while the other 2 developed tumors after 4 weeks (Fig. 7).

## Discussion

Many studies have shown that stromal cells with stem cell potency can be isolated from human umbilical cord mesenchymal tissue, namely, Wharton's jelly [12],[13], and hUC-MSCs are a promising tool for disease treatment. MSCs support the expansion of other stem cells, such as hematopoietic stem cells, are well-tolerated by the immune system, and have the ability to home to tumors [14],[15]. In contrast to bone marrow MSCs, hUC-MSCs have greater expansion capability, faster growth *in vitro*, and more readily available. Although hUC-MSCs have been proven to be therapeutic in several different pre-clinical animal models of human disease, such as neurodegenerative disease, cancer, and heart disease, the effects of their proliferation during long-term *in vitro* culturing remain unclear. Culture of hUC-MSCs is a dynamic process, and improper procedures during this process will result in adverse changes in the cells' inherent properties [16],[17]. Thus, the requirement of cell expansion *in vitro* may cause some specific risks. Several studies on MSCs from different sources have highlighted how genomic instability could lead to spontaneous immortalization and malignant transformation. Spontaneous malignant transformation of mouse BM-MSCs has been described following long-term culturing *in vitro* [18]. Further, some publications have reported spontaneous transformation of hUC-MSCs as well [6],[7],[19]. Further studies are urgently needed in this area to ensure the long-term safety of hUC-MSCs *in vitro*.

In the present study, 10 human umbilical cords were obtained from healthy full-term naturally delivered newborns. The cords were selected according to the America Association of Blood Banks technical manual. Special care was taken not to collect the perivascular cells in the cord (pericytes), which have different characteristics despite being MSCs [20]. MSCs were separated from the umbilical cords. Conventional karyotyping was performed at passage 0 on cells from the 10 donors. If the results at passage 0 were normal, the umbilical cord was included in the study; if not, it was discarded. To objectively compare and contrast study outcomes, we analyzed the morphology, immunophenotype, and multi-lineage differentiation potentials of the MSCs according to ISCT standards. In 2006, the ISCT proposed the following minimal criteria for defining human MSCs [21]: First, the cells must be plastic adherent when maintained under standardized culture conditions. Second, they must express CD105, CD73, and CD90 but not CD45, CD34, CD14, CD11b, CD79a, or CD19, or HLA-DR surface molecules. Third, they must differentiate into osteoblasts, adipocytes, and chondroblasts *in vitro*, as MSCs have multiple differentiation capacities. These standards were adopted by other investigators examining adipose tissue-derived MSCs [22],[23]. In these studies, aside from the surface molecules mentioned above, CD44, CD166, CD80, CD86, and CD4 were also detected. In our study, at the 3rd, 5th, 8th, 10th, and 15th passages, MSCs were tested for osteogenic, chondrogenic, and adipogenic differentiation, and surface antigens were measured by flow cytometry. The expression of surface antigens met the standards set by the ISCT. In a previous study performed by Dah-Ching Ding et al., 10∼16% of human adipose-derived stem cells were positive for the CD34 surface marker, which is often expressed on hematopoietic stem cells and may adhere to the adipose-derived stem cells. The different outcomes among studies could be attributed to differences in cell preparations, culture medium, and the timing and method of isolation. In our study, although the MSCs could differentiate into osteoblasts, adipocytes, and chondroblasts *in vitro*, they showed a weaker ability to differentiate following an increase in the frequency of subculture. In serum-free and in serum-containing conditions hUC-MSC have comparable differentiation potential and immunophenotyping. This finding seems in agreement with the phenotypic and functional properties reported by others[24].Compared between cells of different generations from one donor and between cells of different donors at the same generation. It took different time to harvest P_0_ from different donors (*P<0.05*). After subculture, cells exhibited similar biological characteristics. The population doubling time of serum-containing cultured hUC-MSCs was significantly shorter than serum-free cultured ones (*P<0.05*).

In human chromosomes, telomeres consist of thousands of copies of 6-base repeats (TTAGGG). Telomere length is progressively shortened with each cell division both *in vivo* and *in vitro*, due to the inability of the DNA polymerase complex to replicate the very 5′ end of the lagging strand. The progressive shortening of the telomeres has been proposed as being the main trigger for replicative senescence, because it functions “as an internal clock”, with every cell division the number of telomere repeats decreases[25]. In our study, karyotype analysis by G-band showed no translocation or losing of chromosomes in any MSC groups. The biosafety of hUC-MSCs should be further investigated using molecular karyotyping methods, such as array-CGH, classic cytogenetics, and subtelomeric fluorescence in situ hybridization. Because of the high resolution it affords and in light of the difficulty in obtaining cultured MSC metaphases, array-CGH may be considered as the method of choice for characterizing the genomic situation of MSCs expanding *in vitro*. In the present study, we compared tumor formation in mice injected with early- and late-passage MSCs. These tumorigenicity assays showed that no malignant transformation was noted in mice injected with cells from either passage. Previous studies [26],[27] also showed that MSCs do not spontaneously undergo malignant transformation. There have been few reports on spontaneous human MSC in vitro transformation, of which two turned out to be caused by contamination by tumour cell lines and were retracted afterwards[28]-[31]. In contrast to mouse MSC studies, four of the induced human MSC transformation studies consist of the exogenous expression of human telomerase reverse transcriptase(hTERT) in human cells. This may be attributed to the much shorter telomeres in human MSCs than their mouse counterparts, the much shorter life span of mice than human and the difference in telomere damage signaling pathways between mouse and human. Malignant Transformation Potentials of Human Umbilical Cord Mesenchymal Stem Cells Both Spontaneously and via 3-Methycholanthrene Induction[27].

In conclusion, our data showed that although the differentiation ability of and telomerase activity in hUC-MSCs show a trend of gradual decreasing with culture duration, these cells do not show an aptitude for spontaneous transformation and can be safely expanded *in vitro* in serum-free medium without any sign of immortalization or development of chromosomal abnormalities.
